# Template Entrance Channel as Possible Allosteric Inhibition and Resistance Site for Quinolines Tricyclic Derivatives in RNA Dependent RNA Polymerase of Bovine Viral Diarrhea Virus

**DOI:** 10.3390/ph16030376

**Published:** 2023-03-01

**Authors:** Mitul Srivastava, Lovika Mittal, Debapriyo Sarmadhikari, Vijay Kumar Singh, Antonella Fais, Amit Kumar, Shailendra Asthana

**Affiliations:** 1 Computational Biophysics and CADD Group, Computational and Mathematical Biology Center, Translational Health Science and Technology Institute, NCR Biotech Science Cluster, Faridabad 121001, India; 2 Centre for Biological Sciences (Bioinformatics), Central University of South Bihar, Gaya Panchanpur Road, Bihar 824236, India; 3 Department of Life and Environmental Sciences, University of Cagliari, Monserrato, 09042 Cagliari, Italy; 4 Department of Electrical and Electronic Engineering, University of Cagliari, Via Marengo 2, 09123 Cagliari, Italy

**Keywords:** bovine viral diarrhea virus (BVDV), RNA-dependent RNA polymerase (RdRp), BVDV inhibitor, molecular dynamics simulations, free energy calculations, metadynamics

## Abstract

The development of potent non-nucleoside inhibitors (NNIs) could be an alternate strategy to combating infectious bovine viral diarrhea virus (BVDV), other than the traditional vaccination. RNA-dependent RNA polymerase (RdRp) is an essential enzyme for viral replication; therefore, it is one of the primary targets for countermeasures against infectious diseases. The reported NNIs, belonging to the classes of quinolines (2h: imidazo[4,5-g]quinolines and 5m: pyrido[2,3-g] quinoxalines), displayed activity in cell-based and enzyme-based assays. Nevertheless, the RdRp binding site and microscopic mechanistic action are still elusive, and can be explored at a molecular level. Here, we employed a varied computational arsenal, including conventional and accelerated methods, to identify quinoline compounds’ most likely binding sites. Our study revealed A392 and I261 as the mutations that can render RdRp resistant against quinoline compounds. In particular, for ligand 2h, mutation of A392E is the most probable mutation. The loop L1 and linker of the fingertip is recognized as a pivotal structural determinant for the stability and escape of quinoline compounds. Overall, this work demonstrates that the quinoline inhibitors bind at the template entrance channel, which is governed by conformational dynamics of interactions with loops and linker residues, and reveals structural and mechanistic insights into inhibition phenomena, for the discovery of improved antivirals.

## 1. Introduction

Bovine viral diarrhea virus (BVDV), belonging to the Pestivirus genus of Flaviviridae, is a major viral pathogen in cattle and other ruminants [1,2,3,4,5]. Infections by BVDV can result in a wide assortment of disease manifestations, which include resorption, mummification, or abortion of the dead fetus. In contrast, those fetuses who survive early infection may be malformed or blind, and may have skeletal defects, respiratory problems, underdeveloped brains, or weak immune systems. The result is a high mortality rate in cattle worldwide, which causes significant economic damage due to loss of milk production, reproductive wastage, and increased risk of morbidity and mortality. Indeed, BVDV-related infections are the most expensive viral diseases in many nations’ cattle herds [6].

**Figure 1:**
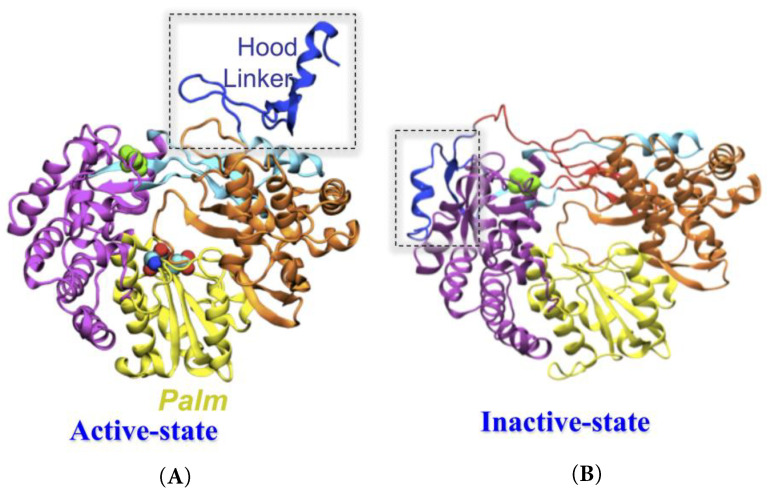
The architecture of BVDV-RdRp in (**A**) active and (**B**) inactive states, shows different conformations of hood and linker. Protein is rendered in new cartoon representation.

Albeit vaccines are effective against BVDV, and cover BVDV infections, additional therapy is required to eradicate BVDV infections [7]. With this aim, a promising option is the use of non-nucleoside inhibitors (NNIs), that specifically target the active form of BVDV RdRp, which is differentiated by the positioning of linker and hood (Figure 1A,B) [4,8]. Following this route, some selective anti-BVDV inhibitors have been discovered [2,5,8,9,10,11,12,13,14,15,16]. However, the molecular recognition of BVDV RdRp with allosteric NNIs has not been extensively studied, as no co-crystallized NNI-RdRp has been published to our knowledge. However, several mutagenic information-based computational docking studies have been reported to date [4,5,17].

Carta A. et al. [13] have reported the synthesis of three new classes of linear N-tricyclic inhibitors, derived by condensation of the quinoline nucleus with 1,2,3-triazole, imidazole, or pyrazine, obtaining triazolo[4,5-g]quinolines, imidazo[4,5-g]quinolines, and pyrido[2,3-g]quinoxalines, respectively. Several derivatives emerged as attractive antiviral agents, endowed with in vitro activity against ssRNA+ viruses. In particular, two selected inhibitors, 2h and 5m, were able to prevent virus-induced cell death and showed potent activity against BVDV in cell-based (EC50 = 1.2 ± 0.07 μM and 11.0 ± 1.1 µM) and in RdRp enzyme-based assays (IC50 = 0.06 ± 0.01 μM and 1.0 ± 0.3 µM) [13]. Despite these exciting findings, two essential aspects have not been addressed. Firstly, the characterization of resistant mutants of the lead compounds (2h and 5m), whose missing information could be the leading cause for the failure of the antiviral drug development pipeline. The second aspect concerns the identification of their binding site and inhibition mechanism. 

We aim to explore the selection of BVDV strains resistant to 2h and 5m, and to analyse their mutational pattern, confirming RdRp as the enzymatic target of inhibition. Intriguingly, imidazoquinoline compound 2h, which exhibited the most potent and selective activity against BVDV, was also active, albeit with modest selectivity, against HCV (Table S1). Compound 2h proved less selective in the replicon assay, because of cytotoxicity directed explicitly against the GS4.1 cells. Moreover, in enzyme assays, compound 2h proved to be 100-fold less potent against the HCV than the BVDV recombinant RdRp, suggesting that, although with different efficiency, 2h targets the viral RdRps.

We adopt a computational, multi-step approach to characterizing the structural and dynamical determinants of the interactions between wild type (WT), and to identify the possible resistant mutant of RdRps for compound 2h. The binding affinity was evaluated through the MM/PBSA approach and compared with experimental IC_50_ values. Apart from conventional molecular dynamics, the enhanced sampling technique, metadynamics, was also implemented, to explore the free-energy landscapes associated with dissociating the inhibitors from their identified binding site. This step helped to identify alternate binding poses of inhibitors while moving out of the binding pocket. Using both approaches, we confirmed the key residues involved in the selectivity of inhibitors, other than the binding site and the structural and functional regions, i.e., structural determinants of the protein involved in ligand recognition. Overall, these results reconcile experimental data with the microscopic information from computational approaches, to explore the quinoline tricyclic derivatives for antiviral development. Unveiling these aspects will provide valuable microscopic information for discovering potent anti-Flaviviridae candidates, and understanding general mechanistic aspects, such as inhibition and resistance.

## 2. Results

A crucial step in studying molecular recognition, is the choice of the ligand’s starting position, which requires two essential pieces of information: (i) the location of the binding site, and (ii) the most likely orientation of the ligand, i.e., its binding mode. Therefore, these two steps were investigated through a multi-step computational protocol, for compounds 2h and 5m. A solvated unliganded protein (hereafter APO) was subjected to MD simulations in a physiological environment. Notably, the average RMSD value of APO, calculated over all atoms to the first snapshot (at time t = 0), was 3.2 Å (Table S2) [18]. Such a considerable value of RMSD suggested that the APO had large structural fluctuations during MD, which in turn might have affected the size and the arrangement of the probable binding site. Therefore, an ensemble of protein conformations (50 ensembles from the last 10 ns of equilibrium trajectory) was generated for the binding site and binding mode identification, by exploiting time-dependent, multi-conformational structural features of APO. This protocol was successfully used in an earlier study to identify the binding site as well as the binding mode of ligands [19].

### 2.1. Identification of the Most Likely Poses of Compounds

Two independent unbiased binding site search methods, based on different levels of theory, were applied. First, AutoLigand (AL) [20] exploited the protein structure alone to predict a binding site, and the consensus sites among all ensembles with high EPV were chosen for further analysis. Second, molecular docking, with the blind docking (BD) approach [21], was performed on the whole surface of the RdRp, to obtain the unbiased mapping of inhibitors. In BD, for each ensemble, the lowest energy inhibitor pose was retained, resulting in 200 best-score orientations per ligand, which were finally ranked and clustered based on selection criteria (see Section 4). The location of the highest scoring sites of 2h and 5m, either obtained from AL and/or the clusters obtained from BD, are shown in supplementary figures (Figures S1 and S2). The largest cluster of 2h contained 52 orientations, with a score of −10.64 kcal/mol, and identified as BSite1, located close to *motif I* in RdRp. The second cluster, with 39 orientations and a −8.16 kcal/mol score, covering a region close to the C-terminal, and the third cluster, with a score of −9.06 kcal/mol and containing 22 orientations, located on the surface of the thumb domain, identified as BSite2 and BSite3, respectively (Table S3 and Figure S1). In the case of compound 5m, the consensus sites, BSite2 and BSite3, differ from 2h. However, the BSite1 was similar to 2h (albeit with a slight difference in binding site residues) (Table S3, Figures S1 and S2). To conclude the binding site identification step, the top three pockets from each method were selected as putative binding sites, and were used for further refinement. The focused study on the three docking sites, revealed that the occurrence of Bsite1 was predominant, as it turned out to be the highest ranked binding site (Figure S3 and Table S4). Nonetheless, this site is also in agreement with the experimental information-based guided docking approach (Table S4). Therefore, BSite1 (including residues found by both approaches), localized in the finger domain and fulfilling the selection criteria: (i) highest docking energy; (ii) larger cluster population; (iii) lowest EPV value from AL; and (iv) vicinity of reported resistant mutations, was considered as the most likely binding site. Furthermore, to assess the stability of compounds at site BSite1, we performed an MD simulation.

### 2.2. Molecular Dynamics Simulations

**Figure 2:**
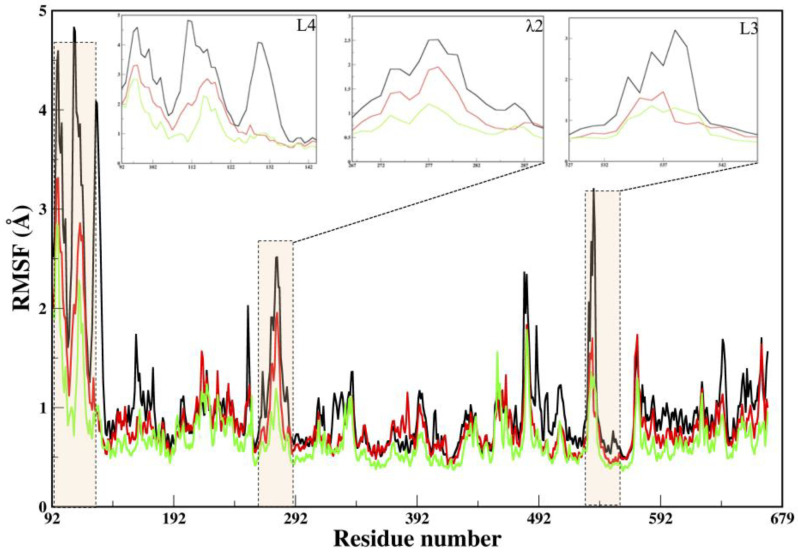
Dynamical characteristics of the Cα atomic fluctuation, represented by RMSF of different systems elucidated through conventional MD simulations. The regions L4, λ2, and L3, showing significant fluctuations, are highlighted in transparent bars. The insets of these regions are shown, to highlight the differences in fluctuation. The color coding of systems is as follows: black (APO), green (COM1), and red (COM2).

MD simulations were further carried out to investigate the structural determinants that govern the target selectivity, and validate the most likely pose of the compounds. To verify the conformational stability of each site for ligand 2h, we calculated RMSDs for MD trajectories (Figure S4). The other two sites (Bsite2 and Bsite3) were found to be unstable, as their RMSDs increased enormously, from an initial 2–4 Å to up to 8–10 Å (Figure S4). Therefore, all the simulations started from Bsite2 and Bsite3, were terminated after 10 ns of the MD simulations. Similarly, for 5m, the pose at BSite1 was found to be stable. Hence, we obtained complexes of 2h (COM1) and 5m (COM2) that were found to be stable at BSite1. A comparison between X-ray (1S48) [22] and the lowest RMSD, from the average structures extracted from equilibrium trajectories of APO, COM1, and COM2, are 2.1 Å, 2.6 Å, and 2.3 Å, respectively, indicating some marginal differences among the systems (Table S5). However, a significant behavior change was noticed at the N-terminal (Figure 2). 

For the measurement of flexibility, RMSF versus the residue number of APO and COMs shares quite a similar distribution (Figure 2). The slightly larger value of RMSF per residue of COM2, may indicate a relatively weaker binding of 5m to RdRp, compared to ligand 2h of COM1; otherwise, both complexes have quite similar fluctuations (Figure 2). The flexibility of APO is much larger than that of the complexes (COMs), especially in the N- and C-terminals and loops of binding sites (L1 and L3) (Figure 2). The different dynamic behavior of the N-terminal is noteworthy, as it is supposed to be involved during polymerization [4,18,19]. This observation is in agreement with the RMSD residue-wise analysis, and can be easily explained in terms of binding between RdRp and inhibitors, that directly reinforced the rigidity of the COMs in those key regions (Tables S2 and S5).

### 2.3. Characterization of Binding Site

**Figure 3:**
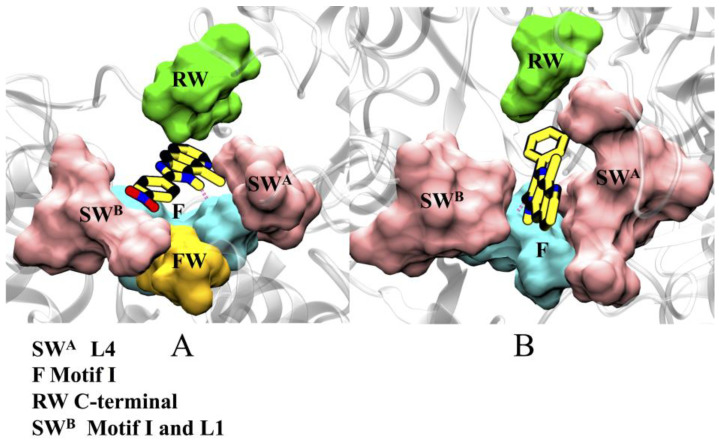
The most stable poses of (**A**) 2h and (**B**) 5m, obtained from MD simulations, reveal their binding mode at the NTP entry site. The regions RW (C-terminal), SW^A^ (L4), SW^B^ (Motif 1 and L1), and FW and F (motif 1), are shown in surf representations, and are colored in green, mauve, yellow, and cyan, respectively.

The compounds, 2h and 5m, were found to be localized at the template entrance site (TES) of RdRp, which is formed by: loops L1 (P388-I398) and L2 (A221-N229) of the finger domain, constituting the side wall A (SWA) of the binding site, loop L3 (L530-G537) of the thumb domain, and L4 (L125-R132) of the N-terminal domain, involved in making side wall B (SWB), motif I (A260-E265) of the fingertip region, which flanks the cavity, constituting the floor (F), and residues T160, D161, and T172 of the finger domain, making the front wall (FW), while Y674 of C-terminal, constitutes the rear wall (RW) (Figure 3). The residues lining the inhibitors are mostly hydrophobic (A221, A222, F224, I261, P262, I287, and A392) in nature, which is consistent with the chemical properties of the inhibitors. Other residues of the binding site are polar (T160, T162, N217, N264, S532, and S533); three basic (R127, R130, R132), and two acidic (D126, E128) residues are present. Some residues, mainly from motif I: I261, P262, N264, and L3: A392, displayed favorable common interactions, indicating a common binding site of both inhibitors (Table S6).

The structures displaying the lowest RMSD from the averages, were extracted from equilibrated dynamics of the COM1 and COM2, and were subsequently used as starting structures for the metadynamics simulations. Dissociation-based free energy calculations were used to explore the key determinants, the binding affinity of inhibitors, and the possibilities of locating alternate binding pockets during dynamics, with an enhanced sampling method, which is rarely feasible in docking (due to static structures, solvent exclusion, and lack of backbone flexibility of protein residues), and/or in MD (due to a timescale problem). The free energy simulations were performed as a function of two collective variables, which have been previously reported to characterize molecular recognition processes [23] (see Section 4 for detail).

The free-energy landscape of the undocking process provides the stable state of complexes in the form of the deepest minimum. The occurrence of more than one minimum could be an alternate binding site, which might be involved in slowing down the undocking (dissociation) procedure, and vice versa could be a reason for an increased residence time of the inhibitor [24,25,26,27,28]. Dror O.R. et al. [28] reported that slow unbinding leads to long drug—receptor residence times, that can dramatically enhance therapeutic efficacy at equivalent affinity. Apart from performing a comparative analysis of the minima obtained, to determine the most stable and biologically relevant binding poses, this approach can also help to guess the putative undocking path of inhibitors. Detailed descriptions are discussed in the following subsections [29].

### 2.4. Protein Complexed to Ligand 2h (COM1)

**Figure 4:**
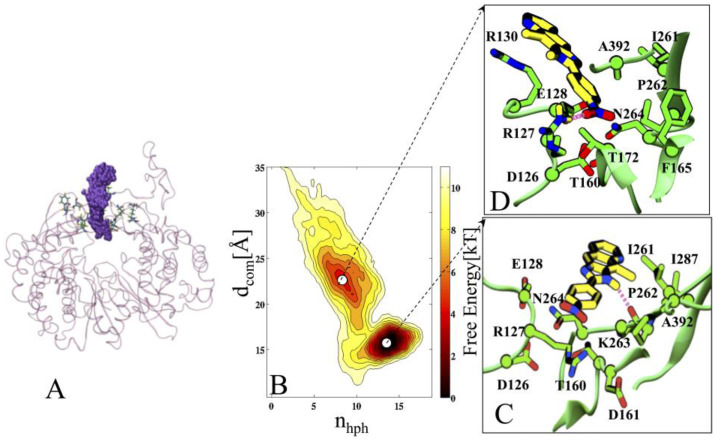
Characterization of 2h unbinding during metadynamics simulation. (**A**) The starting state of 2h. (**B**) the two-dimensional free-energy surface (FES) of 2h in COM1 and its dissection according to the minima obtained during the unbinding process. Each color and each line of FES correspond to 1 kcal/mol. (**C**,**D**) Conformations representing the different states of 2h in COM1, enlarged from two respective basins (mini-1 and 2). The FES of 2h is ~10 kcal/mol. The free energy is contoured in steps of 2 kcal/mol up to 10 kcal/mol. In panels (**C**,**D**), the inhibitor 2h is represented in yellow and amino acids are shown in green. The red dotted line represents the HB between residues and 2h.Gb.

A deep inspection of the free-energy profile of the COM1 unbinding process, indicates that the poses of 2h are clustered around two minima; mini-1 and mini-2 (Figure 4). The mini-1 corresponds to the energetically most stable pose of 2h (Figure 4B).

**Figure 5:**
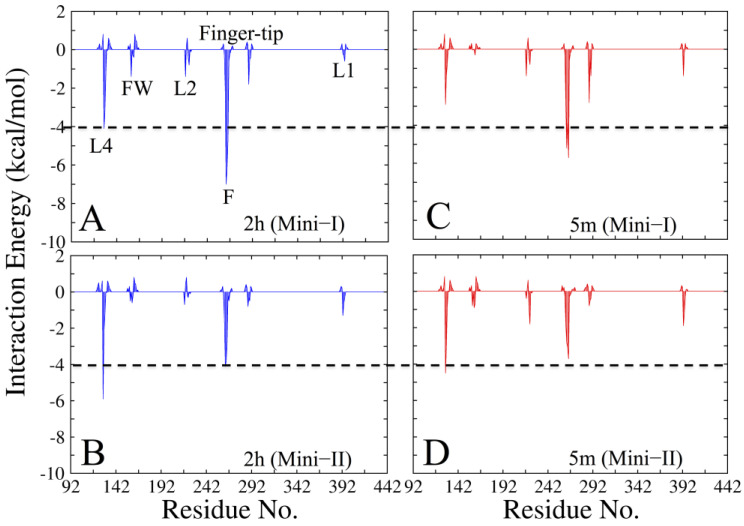
The interaction energy of (**A**,**B**) 2h and (**C**,**D**) 5m in mini-1 and mini-2, respectively.

To quantify more accurately the interaction patterns and the structural determinants of 2h poses during undocking, further 10 ns unbiased standard MD simulations were launched, by extracting the snapshots from each minimum. Ligand 2h stabilizes itself into the cavity in such a way that the N2 atom of its azole moiety makes a strong HB with the P262 of motif I (average dynamic length (ADL) = 2.6 Å), which persists throughout the simulation with occupancy of more than 80% (Figure 4C and Figure 5A). The evolution of the center of mass of 2h during the dissociation process is shown in Figure 5A. The interaction energy analysis revealed that the significant residue-wise contribution comes from R127 (−8.4 kcal/mol) of loop L4 and P262 (−7.1 kcal/mol) of motif I (Figure 5A and Table S6). The residue-wise contact area analysis has shown the maximum contacts of 2h with residues I261, P262, and I287 (Table S7), to be in agreement with the interaction energy outcomes. The metadynamics simulation suggested that ligand 2h moved from the mini-1, and while exploring the binding site, it localized into another minimum (mini-2). The mini-2 is comparatively less profound than mini-1 (Figure 5D). In mini-2, the drop of binding affinity could be due to the loss of 2h interactions with motif I residues (mainly I261, P262, and K263), and vice versa; an increased affinity was also observed with terminal residues of loops L1 and L4 (Figure 5B and Table S6). In particular, an HB was established between L4@R127 (guanidinium group) and 2h@NO2 (nitro group), with occupancy >65% and ADL = 2.8 Å (Figure 4D). Interestingly, this HB was weaker in mini-1, where its occupancy was <40%, and ADL = 3.2 Å. The further establishing factor was the formation of another HB between RW@T160 and 2h@NO2 (nitro group), with occupancy of 54% and ADL = 3.1 Å. In addition, ligand 2h was further stabilized by HpH interactions with residues I287 (fingertip) and A392 (loop L1). For ligand 2h to escape from mini-2, it would require an unbinding energy barrier of ~7 kcal/mol, indicating good thermodynamic stability of 2h in mini-2 (Figure 5B). A closer inspection of the free-energy profile revealed that ligand 2h required ~11 kcal/mol energy barrier to exit from mini-1 to the solvent environment (for complete escape) (Figure 4B). The two considerably deep minima confirm the higher thermodynamic stability (high affinity) of 2h.

The effect of the ligand undocking was analyzed by calculating the average minimum distance between the binding site loops, from unbiased MD simulations of each minimum (Table 1). This result showed that the distances centered differently in each set of loop pairs; in particular, the significant variation of distance distribution was observed in loop pairs L1–L4 (centered around ~14.3 Å in mini-1 and ~22.1 Å in mini-2) and L2–L4 (centered on ~19.6 Å and ~26.3 Å in mini-1 and mini-2, respectively). These values between loop pairs put forth an opening of the binding site during dissociation from mini-1 to mini-2 (Table 2). Subsequently, the first shell water analysis (water molecules ~2.5 Å around the inhibitor) showed a higher number of average water molecules in mini-2 (eight water molecules) compared to mini-1 (five water molecules). Thus, confirming the opening of the binding site, as more water molecules can enter into the binding site. Another piece of evidence regarding the pocket’s opening is evident from the area analysis, as the area distribution ranges from 32.1 Å in mini-1, to 53.2 Å in mini-2. These outcomes show the dynamic nature of the binding site, which was restricted in the presence of inhibitors as the inhibitor moved from the most stable binding site, mini-1, to mini-2, and became a possible reason for RdRp regaining its dynamic nature.

### 2.5. Unbinding Mechanism and Escape of Ligand 2h

The backbone atom RMSD between the binding site residues of mini-1 and mini-2 was 1.3 Å, which reflects a local conformational change of the binding site from mini-1 to mini-2. During unbinding, a rotation of 2h occured around its inertia axis, by which it moved from mini-1 to mini-2. The first event observed in the unbinding mechanism was the breakage of intra-HB between P262@M1 and N2@2h, thereby leading to the detachment of the azole moiety from motif I (Figure 4C). Due to this reorientation, the phenyl group of 2h was stacked against the guanidine group of R127. This stacking further strengthened the HB formed between the R127@L4 and O2@2h, increasing occupancy by more than 50% in mini-2 compared to mini-1 (Figure 4D). The loss of contact with residues of motif I and loop L2 in mini-2, increased the flexibility of the binding cavity. Therefore, ligand 2h started moving from the mini-2 to the solvent environment. The electrostatic interaction between 2h@NO2 (nitro-group) and residue L4@R127 (guanidine-group) was the last interaction to be lost, and afterwards, ligand 2h moved out to a more solvent-exposed region. Finally, all the interactions of 2h with the protein disappeared, and with the increased flexibility of the L4 and L1 loops, ligand 2h escaped into the solvent through the template entrance site.

### 2.6. Protein Complexed to Ligand 5m (COM2)

**Figure 6:**
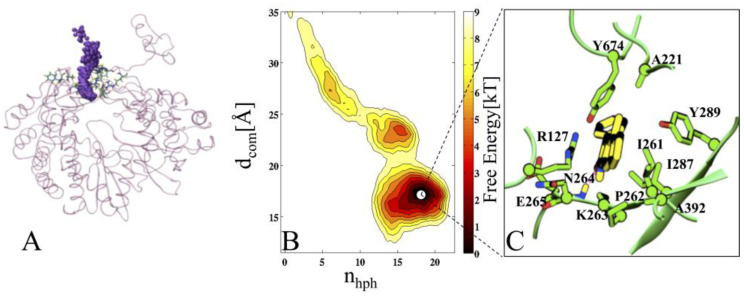
Characterization of 5m unbinding during metadynamics simulation. (**A**) The starting state of 5m. (**B**) The two-dimensional free-energy surface (FES) of 5m in COM2, and its dissection according to the minima obtained during the unbinding process. Each color and each line of FES correspond to 1 kcal/mol. (**C**) Conformation representing the state of 5m in COM2, enlarged from mini-1. The FES of 2h is ~10 kcal/mol. The free energy is contoured in steps of 2 kcal/mol up to 10 kcal/mol. In panel (**C**), the inhibitor 2h is represented in yellow, and residues acids are shown in green. The red dotted line represents the HB between residues and 5m.Gb.

In-depth exploration of the FES of the unbinding process, indicated the different poses of 5m, mini-1 (the deepest minimum), and a less stable mini-2. A separate study (additional unbiased 10 ns MD simulations from each minimum) was performed to generate the key interaction map, and to analyze the dynamical and structural properties of 5m during its undocking mechanism (Figure 6A–C).

In the interaction map of mini-1, the main stable factor of 5m was the formation of two hydrogen bonds (Hbs): First, N3@5m and O1@P262 (ADL = 1.9 Å), and second, O1@5m and HN@N264 (ADL = 2.2 Å), with its pyrido moiety. Another stabilizing factor was hydrophobic (HpH) contacts, mainly with residues A392 (loop L1), and I261 and P262 of motif I (Figure 6C and Table S6). Additional stability of 5m was observed in terms of electrostatic contributions, mainly provided by residues P262 and N264 (Table S5). The per residue contact area of 5m with RdRp residues agrees with the interaction energy analysis (Figure 5C and Table S6). The evolution of 5m from the most stable pose to the external solvent environment was similar to 2h (Figure 6A). In mini-2, an HB formed between N3@5m (pyrido moiety) and O@Thr160, with ADL = 2.3 Å. The phenyl tail of ligand 5m tilted from its mini-1 pose, and in this conformation formed HpH contacts with A221, F224, and L225 residues of the L2 loop. However, at this stage, the major unstabilizing factor was the complete loss of HBs with the motif I residues P262 and N264, and HpH contacts with I261, which led ligand 5m to move from mini-2 (Figure 6B,C). The energy required to move 5m from mini-1 to the solvent environment, implied a barrier of ~9 kcal/mol (Figure 6B).

### 2.7. Unbinding Mechanism and Escape of Ligand 5m

The primary step of the unbinding process was the detachment of 5m from the motif I residues, by breaking HB with N264, and the loss of HpH contacts with I261. Due to the loss of interactions with the motif I residues, ligand 5m moved from its original axis, and this instability imposed the ligand on moving from mini-1 to mini-2. The phenyl ring made HpH contacts with loop L2 residues at this stage. From the mini-2 to the complete exit of ligand 5m, it was mainly guided by the terminal residues of loops L1 and L3, and finally with loop L4. In order to reveal the conformational changes of the binding site from mini-1 to mini-2, the minimal distances among loops were carried out, and a considerably large distribution between loop pairs (especially L1–L4 and L2–L4) was sampled in mini-2 compared to mini-1 (Table 1). Additionally, a similar finding was obtained from area analysis and first shell water analysis, indicating the opening of the binding site in mini-2 rather than mini-1. Ultimately, it lost all interactions with RdRp residues and reached the solvent.

From the metadynamics simulations, we have drawn some clues about the specific activity of the inhibitors apart from energetics, such as: (i) the location of the deepest minimum might affect the specific activity of the RdRp, (ii) it provides some rationale which could be essential for further modification of the inhibitors, with respect to their key residues, which was unveiled during the path analysis, (iii) it was noticed that 2h moved in between loops L3 and L4, while 5m moved out between loops L1 and L4, indicating that loop L4 (linker) is the primary key determinant in its stability as well as in the dissociation of inhibitors, and (iv) loop L4 showed its increased flexibility during the time evolution of the undocking process, in agreement with the previous studies of Choi et al. (2006) [30]. Overall, the dynamical details at the full-atomic representation of complexes, through MD, followed by metadynamics, offered a good opportunity to extract the basic essential information for understanding the molecular recognition process.

Once the best pose of inhibitors was verified as a quantitative and established method, MM/PBSA was performed to predict association binding free energy. Since in the complexes, the minimum energy pose obtained from metadynamics corresponded to the stable pose of MD, the MM/PBSA was carried out from the snapshots (800) taken from the last 10 ns MD of COM1 and COM2.

In the MM/PBSA analysis, the total free energy of binding into electrostatic, VdW, and solute–solvent interactions could be separated, thus gaining additional insights into the physics of the inhibitor–RdRp association process. The electrostatic (ΔE_ELE_) and VdW (ΔE_VdW_) terms provided a significant favorable contribution to the binding of the inhibitors, whereas, owing to the polar character of inhibitors, the desolvation penalty paid by these molecules upon binding was quite substantial, which is evident by unfavorable polar solvation energies (ΔG_NP_). Further insight into the forces involved in complex formation can be obtained by analyzing the electrostatic (ΔG_Etot_) and nonelectrostatic (ΔG_NPtot_) contributions (Table 2).

The electrostatic contribution generally disfavors the docking of the ligand to the receptor molecule, because the unfavorable change in the electrostatics of solvation was mainly, but not entirely, compensated by the favorable electrostatics within the resulting ligand–receptor complex. Indeed, from Table 2, we can appreciate that, despite the favorable electrostatic energies in the gas phase (ΔGE), which are −4.5 kcal/mol and −4.6 kcal/mol, the contribution of the polar solvation energies to binding are 24.3 kcal/mol and 22.9 kcal/mol, for ligands 2h and 5m, respectively. The sum of ΔG_EL_ and ΔG_PB_ was not in favor of binding. Table 2 also suggests that the net result of nonelectrostatic interactions (ΔGNP), i.e., the sum of ΔG_vdw_ and ΔG_NP_, was favorable for the formation of the complexes.

Allostery is a purely thermodynamic phenomenon, in which a binding event leads to the loss of freedom of motions of the binding partners, including their internal motions; thus, it is entropy-unfavorable. As shown in Table 2, the initial ΔG_bind_ values (excluding solute entropy) for 2h and 5m are −28.6 kcal/mol and −26.6 kcal/mol, respectively, and one can see that these values are far from the final ΔG_bind_. The solute entropy contribution calculated by the normal mode analysis was very similar for 2h and 5m, and it significantly reduced the estimated binding free energy to −10.8 and −8.9 (Table 2). The results herein are encouraging, as a good agreement between the calculated binding free energy and experimental data is obtained.

### 2.8. Possible Mechanism of Inhibition

The genotype of in vitro selected ligands 2h and 5m resistant BVDV was determined, to unravel the mechanism of antiviral activity. The binding site of 2h and 5m was formed by functional regions of RdRp: namely, motif I*,* loops, and N-terminal, whose involvement is crucial for the active form of the enzyme [18]. Among them, the N-terminal domain is the most peculiar, being exclusive in the RdRps family [18]. Although no clear evidence for the role of this domain has been reported yet, it is believed that the N-terminal, along with the fingertip region, forms the template entrance channel [31,32]. The flexibility of loop L4 (linker) of N-terminal regions is very important for template translocation during polymerization [4,9]. The fingertip domain is believed to be involved in the template/product translocation, dimerization of RdRp within the Replication Complex (RC), or protein–protein interactions, enabling the assembly of an active RC [22,30]. Moreover, the fingertip region contains the polymerase motifs I and II, which are involved in RNA template and nucleoside triphosphate (NTP) binding, respectively. Motif I has been reported to be located near the initiation NTP binding site, and to bind with incoming NTP [22].

Finally, we postulate the possible inhibition mechanism: (i) dramatic conformational changes in loops, specifically, the linker could reduce the area of the template entrance channel (Table 1), (ii) occlusion of the inhibitors close to motif I could create an obstacle to the binding of incoming NTPs (Figure S5), (iii) binding of inhibitors at the template entrance site could interrupt the intrinsic functional flexibility of the linker, which is thought to drive the template into the catalytic site during polymerization, (iv) binding of 2h and 5m at the surface of the RdRp, could hinder the protein–protein interactions and stability between the different nonstructural proteins that compose the replication complex.

### 2.9. Mutation in Hot-Spot Residues Most Likely Drives the Drug Resistance

**Figure 7:**
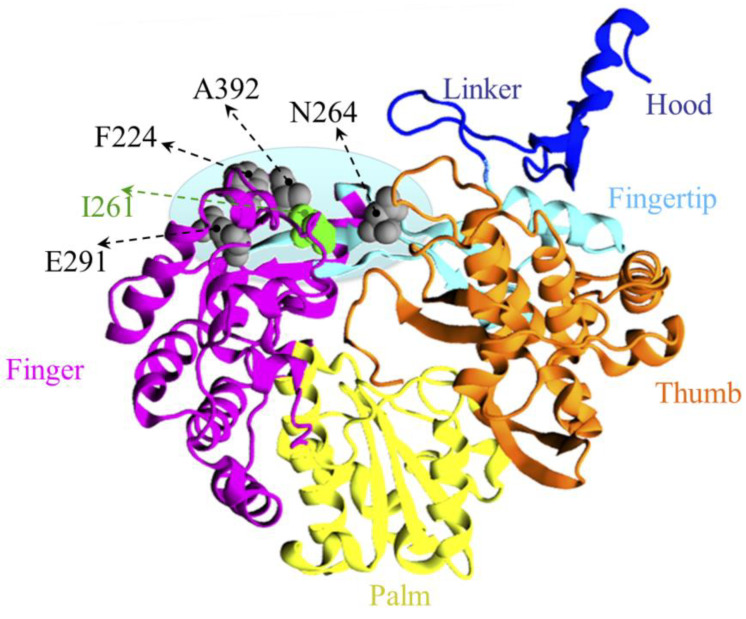
The mutational perspective of BVDV-RdRp. The palm, thumb, and finger domains are shown in the new cartoon representation, and colored in dark yellow, orange, and purple, respectively. The N-terminal and fingertip regions are shown in blue and cyan, respectively. Resistant mutations of different classes of NNIs, residues F224 (in loop L2), E291 (near the fingertip), N264 (in motif I of the fingertip), A392 (in loop L1), and I261 (in motif I of the fingertip) of 227G, are rendered in VDW and colored in gray and green, respectively. The location of resistant mutations is indicated by black arrows.

The emergence of drug-resistant mutations, is the bottleneck in discovering antiviral drugs; therefore, exploring the possible residues that can render the protein active against newly discovered antivirals, is essential. Since the resistant mutations generally occupy the critical zones essential for the function of viral growth, it is pivotal to know the hub zone of occurrence of resistant mutations, such as the thumb and finger domains in HCV RdRp [33]. Therefore, we curate the resistant mutations of BVDV RdRp. The literature indicates the same kind of *hot spot* in BVDV RdRp [33]; however, as per the available information, it has been thought till now that the resistant mutations are localized in the finger domain only (Figure 7). 

Understanding the hot-spot zones and critical residues is paramount, as they can perturb the drug effects and render the protein resistant against them. Therefore, with this aim, we identify those residues that can perturb the compound’s binding, based on the following:

1. The identification of critical residues contributing to the stability of the most stable state (mini-1);
2. Mutational scanning of key residues;
3. Already reported resistant mutations localized in the binding site.

Interestingly, the identified most likely binding sites of 2h and 5m coincide well with already reported resistant mutants, indicating that quinoline series possibly occupy the template entrance site, as we have reported this site earlier, for the benzimidazole class of compounds. The reported mutation residues, such as F224S/Y [4,5,17], E291G [34], I261M [5], N264D [14], and A392E [16] (Table S8), lead two points to be underscored here: (i) All the reported resistant mutants for different classes of compounds are localized in the vicinity of each other, making the zone a hot-spot; (ii) these mutations are common, even for different classes of drugs—F224S/Y is the resistant mutant for indole, imidazopyridine and pyrimidine-amine (Table S8)—indicating the criticality of these residues. We assume that, by mutating these residues, the RdRp is able to resume its function (possibly with different rates with respect to WT). However, the selection of the respective residue depends on the location of drug binding, and how the particular mutations change the dynamics of the RdRp to render it resistant against the respective drug. Therefore, the residues F224, I261, P262, K263, N264, I287, F291, and A392 were picked for mutational analysis, as they fulfil the above-mentioned criteria. Furthermore, it is also interesting to explore how these point mutations rendered the RdRps inactive against quinoline inhibitors, in terms of their thermodynamic changes. 

**Figure 8:**
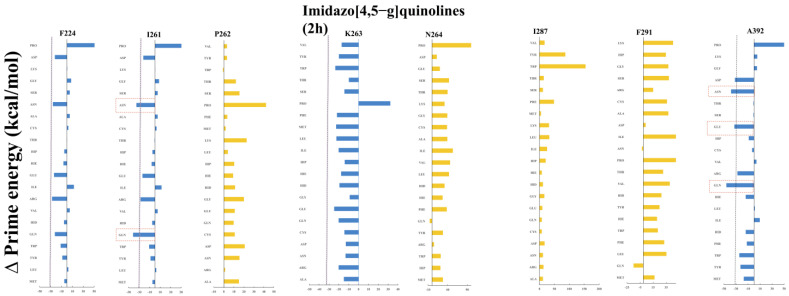
Energy quantification of mutational residues interacting with 2h.

The residue scanning for mutations of key residues was carried out using BioLuminate, which uses the static structure, and only predicts the sidechain rotamer of the mutant residue, followed by a short minimization, by default, in which the solvent effect is treated using a modified MM/GBSA solvent model. Since it is less computationally expensive, the MM/GBSA-based stability predictions were performed for all key residues for the rest of the 19 mutational changes of amino acids (Figure 8).

The mutational analysis indicates that the residues F224, I261, and A392 appear as possible mutational residues for ligand 2h, among key residues (Figure 8). A mutation occurring in these residues might render the RdRp resistant against 2h, underscoring three points:

- All of the reported resistant mutants for different classes of compounds are localized in the vicinity of each other, making the zone a hot spot;
- These mutations are common, even for different classes of drugs, such as F224S/Y, the resistant mutant for indole, imidazopyridine and pyrimidine-amine (Table S8), indicating the criticality of these residues.
- We assume that by mutating these residues, the RdRp can resume its function (possibly with different rates to WT). However, the selection of the respective residue depends on the location of drug binding, and how the particular mutation changes the dynamics of the RdRp to render it resistant against the respective drug. In the case of 2h, the order of possible mutants is A392 > I261 > F224 (Figure 8). The mutational changes suggest that the favorable mutation for 2h could be, in the case of 392 aromatic/acidic/Gln/Asn, for residue 261 Gln/Glu/Arg, and for residue 224 Gln/Asn/Asp/Arg/Tyr. For 2m, the order of preference is I261 > A392 > F224.

**Figure 9:**
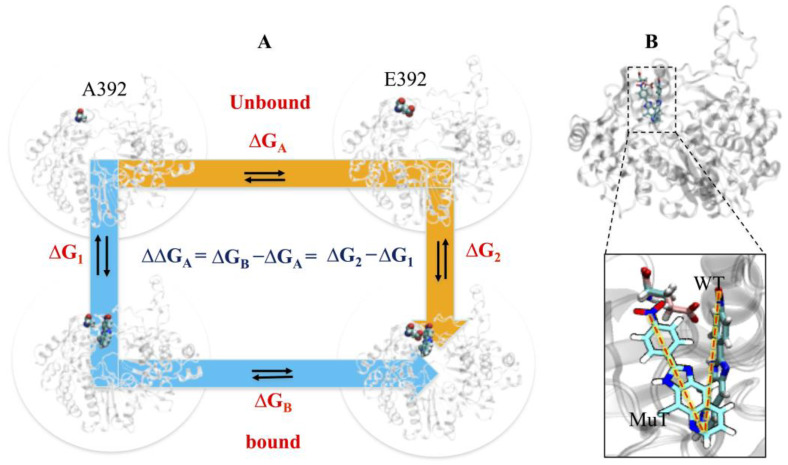
(**A**) Energy calculations of mutant systems via two routes, bound and unbound, respectively. ΔG_A_ represents a change in energy between mutants (A392, E392) in the unbound state, and ΔG_B_ represents a change in energy between mutants (A392, E392) when they are bound to the ligand. ΔG_1_ represents a change in the energy of mutant A392 between bound and unbound ligand states, and ΔG_2_ represents a change in mutant E392 between bound and unbound ligand states. (**B**) Change in the binding mode of 2h observed in the mutant state.

Two-route energy calculations were carried out on the mutants (Figure 9A). In (i), introducing the mutation in the WT protein, to make it MuT, and performing the docking of compounds, to establish ComMuT1. Additionally, a separate ensemble-based docking study was carried out, to highlight the differential sensitivity of inhibitors on wt and mutant RdRps. We observed a considerable loss of pose selection criteria, indicating a less favorable docking in a mutant with respect to WT (Figure 9B). In (ii), performing the docking of compounds in the WT, to establish the complex first, and then introducing the mutation to establish ComMuT2. In the case of 2h, when we mutated 392 from A to E (as it is already reported to have a mutation at the 392 position), then the same WT binding pose failed to be achieved, indicating that the mutation is not allowing the most favorable pose of 2h, possibly due to the long sidechain of Glu (E), compared to Ala (A), at the 392 position. The thermodynamic difference might be a reason for its instability; however, we are not ruling out other possibilities of some other amino acid substitutions. Further, the comparative analysis between WT and ComMuT2 of route 2: Here, we introduce the mutation in WT-com and calculate the ΔG to determine whether they are favorable changes or not.

**Figure 10:**
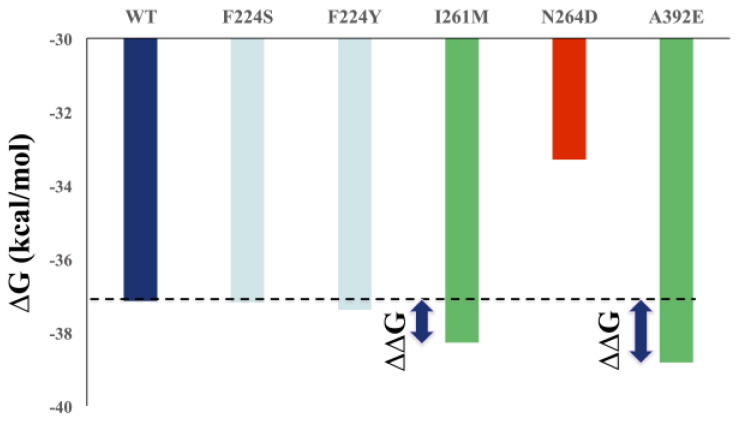
Comparison of binding free-energy values of ligand 2h against WT and mutants.

Increased stability was noticed in the case of mutants of E392 compared to WT (Figure 10), indicating the possibility that the presence of 2h seems not to perform the inhibitory act, and possibly allows the protein to regain its native state for biological function. Since residue 392 is localized in the loop, which is in the vicinity of the linker at the 2h binding pose, therefore, it is possible that intrinsic flexibility is responsible for escorting the RNA template towards the catalytic site. Moreover, the dynamic nature of critical determinants (L1 to L4 loops), that disappear with ligand 2h, allows it to either escape or relocalize into another site, which has minimal or no effect on the drug. Therefore, the viral protein is able to restore its biological function, as a similar event was observed with benzimidazole [4].

The electrostatic effects of the WT and mutants’ complexes were investigated by computing the electrostatic potential grids, by solving the numerical solution of the linearized Poisson–Boltzman equations, and mapped onto the solvent-accessible surface of the binding pocket. As shown in Figure S6, the A392E mutation was found to perturb the electrostatic potential inside the binding pocket. The electrostatic potential surface of the bound 2h is somewhat more similar to COM1 compared to COM^A392E^. The electrostatic repulsion between the nitro group (-NO2) of 2h, and the carboxyl group (-COO) of E392, is the major mutational effect. As is evident in Figure S6, while in COM1, ligand 2h fits comfortably into the binding pocket, and the nitro group is involved with the guanidium group of R127 by making HB. In the ligand 5m, no such electrostatic changes were observed.

The binding affinity between WT and mutant complexes was evaluated through interaction energy, per residue contact area, and binding free energy. The interaction energy difference between COM1 and COM^A392E^ is 3.8 kcal/mol (Table S6). The per residue contact area analysis also reveals a similar trend between WT and mutant complexes, as the difference is 4.9 Å2 (COM1–COM^A392E^) (Table S7), indicating a considerable loss of binding affinity in mutants. 

The correspondence between the estimated free energies of binding, and the experimentally measured IC_50_ values, is one of the benchmarks of molecular recognition [18]. We find a good agreement between the calculated IC_50_ values and the corresponding biological activity determined for two inhibitors in the BVDV-infected cell line (Table 2). We observe that these inhibitors’ rank in their activity towards RdRp is maintained. COM^A392E^ showed a substantial decrease in both electrostatic and VdW energies (Table 2). Overall, the calculated binding free energy difference for the mutant complex (COM^A392E^) was lower than that of WT complexes (COM1 and COM2), indicating that the mutant RdRps are not in identical conformations to WT RdRps (Table 2). Moreover, the mutation A392E affects the binding site and weakens the favorable interactions reshaped to the WT-binding site.

The multifaceted analysis clearly shows that the binding site of mutated RdRps is changed compared to its counterpart. Therefore, it may be easier for a substrate to adapt a geometrically changed binding site in mutated RdRps, to retain its biological functionality, because of its high flexibility. Moreover, it is difficult for less flexible ligands. Thus, we hypothesize that the conformational transformation in the binding site, and the inability of the inhibitors to adapt to the changed binding site in the mutated RdRp, are the reason for drug resistance. 

These regions are very flexible, due to intrinsic requirements, as they are responsible for escorting the RNA template towards the catalytic site. Furthermore, due to their dynamic nature (like linker), as the critical interactions break, they allow the drug to either escape or relocalize into another site, which has minimal or no effect on the drug and, therefore, the viral protein is able to restore its biological function.

## 3. Discussion

Antivirals against BVDV are an unmet need, to counterbalance animal economic burden and mortality. Therefore, not only the discovery of antivirals, but the understanding of existing molecules at the molecular level, is pivotal. Previously, we explored the benzimidazole class of compounds. In a series of publications with the same aim, we are exploring already reported selective and potent antivirals belonging to the quinoline series of NNIs; imidazo [4,5-g]quinolines and pyrido [2,3-g] quinoxalines. Both molecules (2h and 5m) were reported to be active against BVDV RdRps at a low micromolar range. In particular, compound 2h is interesting to explore, as it is potent against BVDV, and substantially active against HCV. However, 5m was reported as toxic in cell lines.

Furthermore, an extensive analysis was also carried out to identify the possible resistant mutant of 2h, that also tried to explore the possible mutants occurring in the RdRp enzyme that could be responsible for acquired resistance. This led us to confirm RdRp as an enzymatic target of inhibition, and obtain helpful information for applying combined experimental and molecular modelling strategies to identify the most likely binding site, and their molecular recognition process. The template entrance site in the finger domain is recognized as the possible binding site of these compounds. We observed a significant conformational flexibility took place in the WT protein, which was limited due to inhibitor binding. Also, a much different dynamic behavior was observed in complexes. 

The parallel implementation of two efficient and predictive binding site detection programs, followed by rigorous docking steps, allowed a mutual validation of the predicted binding pose of ligands, and highlighted the binding site specificity. The identified binding geometries (NNI’s docked pose) were subsequently validated by MD and metadynamics simulations. Thereafter, a comparative analysis between calculated free energy (MM/PBSA value) and experimental IC_50_ was conducted. This analysis provides an improved basis for structure-based ligand design, and provides a possible explanation of the mechanisms of these quinoline series of NNIs. 

Using enhanced sampling techniques (i.e., metadynamics), we can show inhibitors’ alternate metastable binding poses, other than their initial most stable poses. The deeper minimum of 2h indicates its higher affinity to RdRp, compared to 5m. The finding of more than one deep metastable state of 2h, reveals its slow dissociation rate from the binding site and its longer residence time than 5m. The trend of calculated binding affinity obtained from different methods, docking energy, interaction energy, dissociation free energy (metadynamics), and association free energy (MM/PBSA), is in agreement with the corresponding experimental IC_50_ values of both the inhibitors (Table 2 and Table 3).

This is an encouraging performance, and validates the models and molecular modelling studies performed without the co-crystallized structure of the RdRp with NNIs. Interestingly, two previously reported findings: (a) Niyomrattanakit et al. [35], have reported the binding site of anthranilic acid derivatives localized at the template entrance (RNA tunnel) in dengue virus [33]; (b) Dorothy et al. [36], reported, through structure-based phylogenetic analysis, that homomorphs (including most conserved motifs from A to G) linked the template and NTP entry tunnels to the exterior surface of RdRp. Therefore, a complete passage for template and NTP entries are required for the proper functioning of RdRp, with the involvement of structurally and functionally conserved homomorphs. Thus, the occlusion of the template, and/or NTP entry sites, and/or unavailability/hindrance of functionally crucial motifs for RNA template or NTPs, by novel leads could inhibit the RdRp, which is in good agreement with our results.

## 4. Methods

### 4.1. Structural Retreival and Preparation of RdRp and Ligands

X-ray crystal structures of BVDV (PDB ID:1S48) [22] and RdRps was used as a starting model for molecular simulations of the apo-enzyme (hereafter APO). Further, to probe how point mutations would affect the structural and dynamical properties of RdRp, an additional setup for mutant forms of RdRp was constructed by replacing Ala392 with Glu392 and I261 with M261 and T261 residues, in the relaxed, close-to-average structure of APO, by using Maestro (keeping the same orientation as APO). All structures were prepared using Maestro’s Protein Preparation Wizard module (Schrödinger Release 2020-1) [37]. The hydrogen and bond orders were added using PRIME. The hydrogen bond (HB) optimization and restrained minimization were also performed for the systems using the OPLS3 force field model [38,39]. Further, the ligands 2h and 5m were prepared using Ligprep (LigPrep; Schrödinger, LLC, New York, NY, USA, 2022-1), and their optimization was performed using the OPLS3 force field.

### 4.2. Molecular Docking for Site Identification of Compounds 2h and 5m

The identification and characterization of binding sites is a cornerstone of the structure-based drug development process; therefore, extensive docking was carried out using two different methods.

#### 4.2.1. AutoLigand (AL)

AL [20] identifies binding sites based on the potential affinities of probe atoms (C, H, O) for the target protein, and reports the optimal volume, shape, and best atom types for the identified ligand binding sites. In particular, the program searches for the ten non-overlapping points with the highest potential affinity, and fills the cavities starting from these points. Each point (or “fill”) is ascribed an atom type, and each ensemble of points (representing the negative image of a pocket) is assigned an energetic value (Energy/Volume, EPV). The pocket with the lowest EPV is a protein’s most likely binding site. To examine the full RdRp, grids were centered on the center of mass of the protein, and built with 126 × 126 × 126 points, a spacing of 1.0 Å, and a smoothing value of 0.5 Å. The protein cavities were covered from a minimum number of 100 fills to a maximum of 500, using an increment of 50 points. The volume of these fills represents the negative image of a binding pocket, as identified by the AL algorithm. In an attempt to identify cavities present in all MD snapshots, further analysis was performed. Firstly, only pockets with a volume higher than 360 Å^3^ were selected. Then, all residues within a distance of 2.0 Å from fills were identified as “pocket-lining-residues”, to obtain the respective image of pockets containing those fills. Finally, the frequency of such residues was computed, and the cavities shared among all snapshots were detected, considering only pocket-lining residues with a frequency higher than 5%. The mean EPV value associated with these cavities was also computed, to compare and select binding sites into RdRp. 

#### 4.2.2. Blind Docking (BD)

BD [19,21] calculations were performed with AutoDock4.0 [40], to get an unbiased mapping of NNIs binding spots. AutoGrid4.0 was employed to build a 126 × 126 × 126 points grid map, covering the whole RdRp (Figure S1), with a spacing of 0.586 Å and centered on the center of mass of the RdRp. The Lamarckian genetic algorithm, using a scoring function based on the AMBER force field, was used for conformational sampling of 2hH and 5mM. For each docking simulation, 200 runs were carried out, with 300 random individuals in the first population, 2.5 × 10^7^ energy evaluations, and 2.7 × 10^7^ numbers of generations. For each run, the lowest-energy ligand conformation was chosen for clustering, according to an RMSD cutoff of 5.0 Å. Clusters were then ranked and clustered based on AutoDOCK energy scores and populations.

### 4.3. Guided Docking (GD)

We also exploited the available experimental information about the location of single-point resistant mutations of NNIs of different classes [41,42]. More specifically, the grid center for guided docking was placed on the mass center of three residues: I261, N264, and A392 [41]. This choice was made after studies which showed that mutations of the above residues confer resistance to 2-phenyl-benzimidazole [41], arylazoenamine [43], and thiosemicarbazone [16], respectively. We used grid maps of 60 × 80 × 80 points, with distances of 0.375 Å from each other along the three Cartesian directions. The Lamarckian genetic algorithm was employed with the following parameters: 10 million generations and energy evaluations; population size of 300; 200 runs mutation rate of 0.02; crossover rate of 0.80; elitism value of 1. For local search, the so-called pseudo-Solis and Wets algorithms were applied from the default parameter [44]. Consensus binding sites, including residues identified by the above-mentioned approaches, were used for focused docking calculations. Finally, the lowest energy poses of compounds obtained from focused docking were used as a center for refinement to get the most favorable pose of compounds by re-docking calculations. The most likely pose of compounds identified, was subsequently used for standard MD simulations (hereafter COM1 and COM2).

### 4.4. Conventional Molecular Dynamics (MD) Simulations

We performed MD simulations with the NAMD software package [45]. The solute was placed within a truncated octahedral box (of edge length 42.9 Å, ensuring a minimum distance of 146.0 Å between any RdRp atom and the edge of the box) filled with explicit water molecules (TIP3P) and counter-ions. In brief, geometry optimizations were carried out with a two-step protocol: (i) up to 10,000 cycles (2000 of steepest descent plus 8000 of conjugate gradient), with harmonic restraint (k = 1 kcal mol^−1^Å^−2^) on non-hydrogen atoms of the solute; (ii) up to 10,000 conjugate gradient cycles with no restraints. Next, heating up to 300 K was achieved, by linearly increasing the temperature within 100 ps of NVT MD, while imposing restraints of 1 kcal mol^−1^ Å^−2^ on non-hydrogen atoms of the solute. The restraints were then released for 100 ps and, as a last step preceding the productive dynamics, 1 ns of NPT MD was carried out, in order to relax the simulation box [46,47,48]. Finally, we performed MD simulations of 10 ns duration, for each APO and COM, in explicit water solution, under the NPT ensemble. Temperature and pressure were regulated at 310 K and 1.013 bar, using a Langevin thermostat (damping constant 5 ps^−1^) [49,50] and the Nosé–Hoover–Langevin piston pressure control [51]. Electrostatic interactions were evaluated using soft particle mesh Ewald schemes, with 1 Å grid spacing and a cutoff of 12 Å, i.e., the same used for Lennard–Jones interactions.

### 4.5. Analysis of MD Simulation Trajectories

Root mean square deviation (RMSD) and root mean square fluctuation (RMSF) were calculated, to characterize distortions and changes in the flexibility of the proteins. Namely, the RMSD per residue was calculated for: (a) the close-to-average RdRp structures in the APOs and COMs (to highlight significant conformational changes due to inhibitor binding), and (b) the close-to-average RdRp structures in the APOs and COMs with respect to their crystallographic structures (to assess the presence of significant structural differences in the MD runs, see Supplementary Material). The minimum distances between the four loops that define the putative binding site were calculated in different loops (L1–L2, L1–L3, L1–L4, L2–L3, L2–L4, and L3–L4). Finally, we estimated the area of the template entrance tunnel during equilibrated dynamics, by considering the triangle defined by terminal residues (Cα) of the loops contributing to the cavity (R127, F224, and A392) (data not shown). Although the area so calculated is clearly an approximation of the absolute value, a valuable indication of the crucial inhibitor’s effects on the structure and dynamics of the RdRps was obtained (see Section 2 and Section 3). The interaction energies between compounds and key RdRp residues were calculated by summing up the nonbonded (Lennard–Jones and electrostatic) terms of the pair-wise molecular mechanics additive function, calculated between the compounds and key residues of the identified putative binding site. Additionally, the per residue contact area of compounds 2h and 5m with RdRp residues, were calculated, by using the platinum server (http://model.nmr.ru/platinum/) (accessed on 26 January 2023) [52] with default parameters.

### 4.6. Metadynamics

The initial close-to-average structures of COM1 and COM2 were used to perform the undocking process, by using an enhanced sampling method, metadynamics. The undocking process occurs on a timescale that standard MD simulations cannot reach with an all-atom representation. We used the metadynamics algorithm [53,54,55,56,57] to overcome this problem, by using the software package NAMD [45]. This algorithm, based on a history-dependent biasing potential, added in a subspace defined by a chosen set of reaction coordinates *s_α_*(*x*) that, is aimed at reconstructing the multidimensional free energy of a given process. At time *t*, the biasing potential, *V_G_*, is given as the sum of repulsive Gaussian functions added, with a frequency 1/*τ_G_*.

(1) $$V_{G\;}\left(s,t\right)=\;w\;\underset{t^{\prime }=\tau_{G},2\tau_{G},3\tau_{G},\ldots \ldots .;t^{\prime }<t}{\sum }\exp\left(-\frac{{\left[s_{\alpha }-s_{\alpha }\left(t^{\prime }\right)\right]}^{2}}{2\delta s^{2}}\right)$$

where *W* is the Gaussian height, and *δs* is the Gaussian width. Due to this potential, the system is discouraged from revisiting the configurations already sampled. Metadynamics not only allows the acceleration of rare events, but also the reconstruction of the free energy FG(*s*, *t*) = −*V_G_*, which is an approximation of F(*s*) in the region Σ(*s*), explored by *s*(x*G*(*t*)) up to time *t* [58,59]. Free energy reconstruction accuracy depends upon the Gaussian parameters *W*, *δs*, and *τ_G_*. Details of the metadynamics algorithm have been previously described [4].

#### Choice of Reaction Coordinates

The choice of reaction coordinates is pivotal to obtaining the best free energy approximation. The CVs used here to describe the dissociation of inhibitors are: (i) the distance (dCMs) between the centers of mass of the inhibitors and the center of the mass of the RdRps. (ii) The number of hydrophobic contacts (*n_hph_*) between nonpolar carbons on the inhibitors and on the bases that it covers in the starting structure, modelled as a coordination number:

(2) $$n_{hph}=\underset{ij}{\sum }\frac{\left[1-{\left(\frac{r_{ij}}{r_{o}}\right)}^{a}\right]}{\left[1-{\left(\frac{r_{ij}}{r_{o}}\right)}^{b}\right]}$$

The parameters *a* and *b* have values of 6 and 12, respectively, while r_0_ = 6.0 Å accounts for the typical carbon–carbon distance (4/4.5 Å), and the thermal motions’ amplitude is 1.5/2 Å. A similar CV has been applied previously [58,59]. The Gaussian parameters were: w ≈ 0.25 kcal/mol (0.1 kJ/mol), δsCMs = 0.5 Å, and δshph = 6, in both cases. The time interval between two successive Gaussian depositions, was set to 0.5 ps in all the simulations.

Free-energy surfaces were calculated as a function of dCMs and nhph. In addition, simulations were performed in which the three CVs were kept active. In every metadynamics run, the dissociation event was seen after a few ns (see Section 2), and occurred following a very similar mechanism, which means that the relevant slow motions of the systems are captured by our CVs. The activation free energies associated with the dissociation of inhibitors, were extracted by stopping the summation over Gaussians just after the complete dissociation of the drug. The free-energy profiles are shown as a function of dCMs and nhph for both cases (2h–RdRp and 5m–RdRp). 

From each identified minimum, we performed additional standard MD simulations (5 ns), to characterize the structural details and the interaction maps of inhibitors with BVDV RdRps. The characterization of interaction maps of inhibitors followed the same criteria as mentioned above. The change in enthalpy energy of 2h and 5m in the different minima identified was calculated by evaluating the nonbonded interactions (VDW + ele) between inhibitors, and all other atoms (protein, ions, water molecules) which were within a cutoff of 10.0 Å from inhibitors. For electrostatic interactions, we adopted the same scheme (soft particle mesh Ewald schemes) [60] as for the simulations. 

### 4.7. MM-PBSA Calculations

To compare the binding free energies of WT (COM1 and COM2) and mutant (COM^A392E^ and COM^I261T^) BVDV RdRps, MM-PBSA calculations [23] were performed on 400 snapshots taken from equilibrium trajectories (one every 50 ps). The binding free energy (Δ*G_bind_*) of each system was evaluated as follows:

(3) $$\Delta G_{bind}=\;G_{COM}-\left(G_{rec}+G_{lig}\right)$$

where *G_com_*, *G_rec_*, and *G_lig_* are the absolute free energies of the complex, receptor, and ligand, respectively, averaged over the equilibrium trajectory. According to the MM/PBSA method, the free energy difference can be decomposed as:

(4) $$\Delta G=\;\Delta E_{\mathrm{MM}}+\Delta G_{\mathrm{solv}}-T\Delta S_{\mathrm{conf}})$$

where ΔE_MM_ is the difference in molecular mechanical energy, ΔG_solv_ the solvation-free energy (including an entropic contribution), and TΔS_conf_ the configurational entropy (including loss of translational and rotational entropy due to binding, as well as changes in the vibrational entropy). The first two terms are calculated with the following equations:

(5) $$\Delta E_{\mathrm{MM}}=\Delta E_{\mathrm{bond}}+\Delta E_{\mathrm{angle}}+\Delta E_{\mathrm{torsion}}+\Delta E_{\mathrm{vdw}}+\Delta E_{\mathrm{electrostatic}}$$

(6) $$\Delta G_{\mathrm{solv}}=\Delta G_{\mathrm{PB}}+\Delta G_{\mathrm{SA}}$$

where E_MM_ includes the molecular mechanical energy contributed by bonded (E_bond_, E_angle_, and E_torsion_) and nonbonded (E_vdw_ and E_elect_) terms of the force field; ΔGsolv is the solvation free energy, which has an electrostatic (ΔG_PB_, evaluated using the Poisson–Boltzmann equation) and a nonpolar contribution (ΔG_SA_ = γΔSA + b), proportional to the surface area (ΔSA). The electrostatic solvation free energy was calculated using the DELPHI program [61], with dielectric constants of 1 for the solute and 78.5 for the solvent. Atomic radii were taken from PARSE, with an additional value of 1.90 Å for phosphorus, while partial charges were taken from the AMBER/GAFF force fields [23]. The electrostatic potential was calculated on a cubic lattice of length equal to 120% of the longest interatomic distance of the protein, using a grid spacing of 0.5 Å. Up to 10,000 iteration steps were requested to reach convergence in energy (using the linear form of the PB equation). The surface area entered in the equation for ΔG_SA_ was calculated using MOLSURF, with γ and b values of 0.00542 kcal/mol Å^2^ and 0.92 kcal/mol, respectively, for use with PARSE atomic radii. The solvent probe radius was set to 1.4 Å.

The solute entropy contribution (-TΔS_conf_) was estimated by normal-mode analysis using the nmode module of AMBER 9.0. For each snapshot, the structures of the complex, receptor, and ligand were first optimized in the absence of explicit solvent, using a distance-dependent dielectric constant of ε = 4r (r is the interatomic distance) to mimic solvent screening. The convergence cutoff on the potential energy gradient was set to 10^−4^ kcal/(mol·Å). 

### 4.8. Bioluminate: Scanning of Possible Substitution to Predict the Resistant Mutations

The residue scanning in BioLuminate [62] was performed through a residue scanning panel in biologics, with a refinement option set to sidechain prediction and backbone minimization, and the cutoff distance was set to 0.0. BioLuminate calculated the net change in the stability of the protein due to the mutation, calculated using the Prime energy function, with an implicit solvent term. Stability is defined as the difference in free energy between the mutant and wild states produced by a single point mutation. Schrodinger module BioLuminate utilizes [62] the MM-GBSA approach, with force field OPLS3e and solvent model VSGB, to calculate the energy. The main objective of conducting the mutational studies was to understand the effects of point mutations on compound binding, and how they alter the structural and dynamic properties of COM1 and COM2. An additional setup for the mutant form of RdRp (A392E) was constructed, through modification of the relaxed close-to-average structure of COM1, by using VMD. A thorough inspection was done to avoid any steric clashes. The same equilibration and production protocols described below for WT, were employed for the mutant system (hereafter COM^A392E^).

## 5. Conclusions

- The detailed insights obtained from our multi-step computational studies revealed two significant outcomes: (i) A common binding site for compounds 2h and 5m, as they share the key residues and are localized at the template entrance site. The binding of compounds at the template entry site occludes the entrance passage, and their significant interactions with loop L2 (motif G), loop L4 (beta-hairpin motif), and motif I (motif F), make crucial motifs essential for biological function unavailable, therefore, causing inhibition. (ii) Three amino acids, I261, P262, and N264, belonging to motif I of the fingertip region, played a significant role in establishing 2h and 5m potency. This supports the development of a pharmacophore model for establishing more potent leads.
- In summary, identifying a new binding site at the template entry channel appears as a “hot spot” for developing broad-spectrum antiviral drugs against infectious RdRps families, which are known to have a common structural and functional skeleton. Furthermore, identifying the most probable resistant mutation against compound 2h, using residue scanning methods, indicates that A392 is the most likely residue to mutate, possibly rendering RdRp resistant against 2h. Based on the reported mutation, there is the possibility of A392E being resistant against 2h, as it makes the mutant protein more stable, and does not allow 2h to enter the binding site. The computationally-derived mechanistic (inhibition and resistance) studies provide precious insights for improving the reported leads’ specificities and inhibitory potencies, and rationalizing the design of effective bioactive inhibitors with low susceptibility to resistance.

## Figures and Tables

**Table 1:** The minimal average distance between the pair loops (L1, L2, L3, and L4), which correspond to the binding cavity, for the last 3 ns of the MD trajectory of respective minima.

Loop Pairs	Ligand 2h		Ligand 5m	
	Mini-1	Mini-2	Mini-1	Mini-2
L1–L2	11.5(1.6)	13.3(1.7)	9.2(1.6)	11.6(1.1)
L1–L3	19.0(1.7)	21.0(1.9)	16.0(1.1)	23.1(1.8)
L1–L4	14.3(1.3)	22.1(1.4)	17.8(1.3)	29.8(1.6)
L2–L3	13.4(1.5)	17.5(1.6)	13.7(1.9)	16.2(2.4)
L2–L4	19.6(1.5)	26.3(1.7)	14.5(2.2)	25.7(1.4)
L3–L4	8.5(1.5)	11.3(1.9)	10.8(1.2)	15.9(1.7)
First shell water				
	5.0(0.9)	8.0(2.1)	9.0(1.4)	13.0(1.7)

**Table 2:** Experimental and calculated binding free energies of compounds 2h and 5m to the BVDV RdRps. Energies are in kcal/mol; IC_50_ values are in µM. The experimental Δ*G_bind_* values are estimated from IC_50_ data with the equation Δ*G_bind_ = RT ln IC_50_* (R is the universal gas constant, T = 298.5 K), while the calculated values are given by the formula: Δ*G_bind_ =* Δ*E_ELE_ +* Δ*E_VDW_ +* Δ*G_PB_+* Δ*G_NP_* − *T*Δ*S_conf_*. The various contributions are also shown. ^a^ Calculated value, ^b^ experimental value.

System	With Ligand 2h	With Ligand 5m
ΔE_VDW_	−30.0(2.7)	−28.3(3.2)
ΔE_ELE_	−18.6(5.2)	−20.2(2.3)
ΔG_PB_	24.3(5.5)	22.9(5.1)
ΔG_NP_	−4.6(0.7)	−4.5(0.19)
PB_tot_	−28.8(3.1)	−26.6(2.8)
ΔG_ELE+PB_	5.7(5.2)	2.9(3.2)
ΔG_vdw+NP_	−34.6(2.8)	−32.8(1.2)
TΔS_solute_	−18.4(2.5)	−18.3(2.8)
ΔG ^a^/ΔG ^b^	−10.6/−9.8	−8.9/−8.1
IC_50_^a^/IC_50_^b^	0.02/0.06	0.2/1.0

**Table 3:** Comparison of various energy values (kcal/mol) and IC_50_ values for RdRp in complex with ligand 2h and ligand 5m.

Energy	Ligand 2h	Ligand 5m
Docking energy	−11.8	−10.5
Interaction energy	−34.8	−29.3
Association energy (MM/PBSA)	−10.6	−8.9
Disassociation energy (metadynamics)	−11	−9.0
Calculated binding free energy	−9.8	−8.1
Experimental IC_50_ (μM)	0.06	1.0
Calculated IC_50_ (μM)	0.02	0.2

## Data Availability

We encourage all authors of articles published in MDPI journals to share their research data. In this section, please provide details regarding where data supporting reported results can be found, including links to publicly archived datasets analyzed or generated during the study. Where no new data were created, or where data is unavailable due to privacy or ethical restrictions, a statement is still required. Suggested Data Availability Statements are available in section “MDPI Research Data Policies” at https://www.mdpi.com/ethics.

## Supplementary Materials

The following supporting information can be downloaded at: https://www.mdpi.com/article/10.3390/ph16030376/s1, Table S1: Class of compounds against BVDV and HCV RdRps. Table S2: The average RMSD and RMSF values of systems under study. The RdRp correspond to whole protein, while BS and BS + NT represent the binding site and N-terminal, respectively. Table S3: Blind docking results of 2h and 5m on BVDV RdRp. All the docking energies are in kcal/mol. Table S4: Focused docking results of 2h and 5m on BVDV RdRp. a: only the top three sites have been chosen; b: total no. of conformers; c: all the energies are in kcal/mol; d: only those residues within the range of 4 Å are shown, from the probe in Autoligand and representative conformers in BD and GD. Table S5: Comparative average RMSD values of different systems under study. Table S6: Interaction energy between ligand and key residue of the binding site. All the energies are in kcal/mol. Table S7: Contact area analysis of residues interacting with 2h and 5m during unbinding. Table S8: Reported mutations for different classes of compounds. Figure S1: Blind docking reveals the clusters of (A) 2h and (B) 5m. The conformations of 2h and 5m are rendered in yellow. Figure S2: Blind docking reveals the clusters of (A) 2h and (B) 5m. The conformations of 2h and 5m are rendered in yellow. Figure S3: The focused ensemble-based docking (FD) on each conformer of protein, targeting the top five consensus sites identified through AL and BD steps. In particular, the top three sites showed considerable binding affinity. The top three clusters of proteins (shown in inset) were used for subsequent MD runs. Figure S4: Validation of binding pose of 2h, identified at sites 1, 2, and 3, through MD simulation of 10 ns; Figure S5: Occlusion of NTP entry channel upon binding of (A) 2h and (B) 5m. Figure S6: Electrostatic potential surface of (A) wild 2h and (B) mutant 2h. Figure S7: Mutational analysis on 5m.
