# Supplementary material for: Template Entrance Channel as Possible Allosteric Inhibition and Resistance Site for Quinolines Tricyclic Derivatives in RNA Dependent RNA Polymerase of Bovine Viral Diarrhea Virus
Source: Pharmaceuticals (Basel). 2023 Mar 1;16(3):376. doi: 10.3390/ph16030376 (PMC10058290; doi:10.3390/ph16030376)
Supplement: Supplementary file 1 [file pharmaceuticals-16-00376-s001.zip › pharmaceuticals-2213650-supplementary.pdf]

Supplementary material for

Template entrance channel of BVDV RdRp appears as possible allosteric inhibition and resistance site for quinolines tricyclic derivatives

Mitul Srivastava[1,$], Lovika Mittal[1,$], Debapriyo Sarmadhikari[1], Vijay Kumar[2], Antonella Fais[3], Amit Kumar[4,*], Shailendra Asthana[1,*]

[1]Translational Health Science and Technology Institute, NCR Biotech Science Cluster, Faridabad, 121001, India.

[2]Centre for Biological Sciences (Bioinformatics), Central University of South Bihar, Gaya Panchanpur Road, India.

[3]Department of Life and Environmental Sciences, University of Cagliari, Monserrato, 09042 Cagliari, Italy niversity of Cagliari, Italy.

[4]Department of Electrical and Electronic Engineering, University of Cagliari, Via Marengo 2, 09123 Cagliari, Italy.

$ equal contribution
***corresponding authors**

Supplementary Tables

Table S1: Class of compounds against BVDV and HCV RdRps

Class of compounds	BVDV RdRp (EC_{50}/IC_{50})	HCV RdRp (EC_{50}/IC_{50})	Year
Acridones	0.5(±0.2)/NR	1.7(±0.2)/NR	2006
Imidazopyridines	0.20(±0.02)/NR	0.20 (±0.03)/NR	2007
Benzimidazole	0.8/0.002	1.0/0.4	2012/2013/2014
Imidazoquinoline	1.2/0.06	3.1/8.0	2012*

Table S2: The average RMSD and RMSF values of systems under study. The RdRp correspond to whole protein, while BS and BS+NT represent binding site and N-terminal respectively.

**<RMSD> (Å)**				
	**APO**	**COM^{1}**	**COM^{A392E}**	**COM^{2}**
**RdRp**	3.2	2.6	1.8	2.7
**<RMSF> (Å)**				
**RdRp**	1.4	0.7	0.9	1.1
**BS**	1.8	1.0	1.0	0.9
**BS+NT**	2.1	1.4	1.0	1.7

Table S3: Blind docking results of 2h and 5m on BVDV RdRp. All the docking energies are in kcal/mol.

Sites[a]	2h			5m		
	**Focused Docking**		Key Residues[d]	**Focused Docking**		Key Residues[d]
	Clust[b]	Dock energy[c]		Clust[b]	Dock energy[c]	
**AutoLigand**						
BSite1	55	-10.6	D126, E128, R130, R132, I155, K219, K263, N264, E265, K266, I287, I289, K525, T528, R529, K530, D531, S532	46	-9.6	Q124, R132, N133, I134, K263, N264, E265, K266, T528, R529, L530, D531, S532
BSite2	32	-8.6	F106, Q107, G108, N109, K120, I134, Y135, L150, E151, K152, L153, I154, V155, R157, K266, W273, V279, V280, L620	28	-8.2	L125, D126, R127, E128, T160, D161, T162, I261, P262, K263, N264, K266
BSite3	18	-7.6	E213, A214, G215, V216, N217, R218, Y582, L669, Q670, G671, K672, H673, Y674, E675, L679	12	-7.3	E213, A214, G215, V216, N217, R218, T299, Y303, V306, K307, Y316, P408, D409, T410, S411, A412, Q670
**Blind docking (BD)**						
BSite1	62	-11.8	E128, R130, R132, K219, K263, N264, E265, K266, I287, I289, A392, K525, T528, R529, K530, D531, S532	51	-10.2	Q124, R132, N133, I134, K263, N264, E265, K266, T528, R529, L530, D531, S532.
BSite2	41	-9.1	Q124, L125, R157, K130, N133, I134, R132, L530, S531, S532, T528, N264, E265, K266	39	-9.3	F106, Q107, G108, N109, K120, I134, Y135, L150, E151, K152, L153, I154, V155, R157,
BSite3	14	-7.9	E213, A214, V216, N217, R218, Y674, E675, H673, K672, G671, Q670, L669, T668	17	-8.6	N217, R218, K219, G220, A221, D531, S532, S533, E535, H578, G671, K672, Y674
**Guided docking (GD)**						
A	68	-10.7	R127, E128, T160, I261, P262, K263, N264, I287, A392	49	-9.4	I261, P262, N264, K263, I287, A392

Table S4: Focused docking results of 2h and 5m on BVDV RdRp. a: only top three sites has been chosen; b: total no. of conformers; c: all the energies are in kcal/mol, d: Only those residues are shown within the range of 4Å from the probe in Autoligand and representative conformers in BD and GD.

Binding sites	Complex with 2h		Complex with 5m	
	No. of Conformers (#)	Docking energy (kcal/mol)	No. of Conformers (#)	Docking energy (kcal/mol)
**BSite1**	52	-10.64	36	-9.54
**BSite2**	39	-8.16	43	-8.07
**BSite3**	22	-9.06	27	-8.38

Table S5: Comparative average RMSD values of different systems under study.

	APO	COM^1	COM^{A392E}	COM^2	COM^{I261T}
**X-ray**	2.1	2.6	2.5	2.3	2.5
**APO**	0.0	2.7	2.4	2.7	2.6
**APO (BS)**	0.0	3.3	3.7	4.1	4.3
**APO (NT)**	0.0	4.9	3.5	4.9	4.1
**APO (BS+NT)**	0.0	4.9	5.2	5.4	5.3
**APO (no BS+NT)**	0.0	2.1	1.4	1.9	2.1

Table S6: Interaction energy between ligand and key residue of binding site. All the energies are in kcal/mol.

Residues	Compd 2h (kcal/mol)		Compd 5m (kcal/mol)	
	COM^1	COM^{A392E}	COM^2	COM^{I261T}
R127	-8.4	-9.2	-5.1	-5.9
E128	-3.1	-2.8		
A221	-1.0	-0.8	-2.4	-1.4
I261	-1.4	-0.3	-4.2	-2.8
P262	-7.1	-6.8	-5.5	-6.4
D264	-4.8	-3.2	-5.6	-4.9
A392	-2.3	-1.2	-1.2	-0.7
Total	-28.1	-24.3	-24.0	-22.1
**COM^W- COM^M**	-3.8		-1.9	

Table S7: Contact area analysis of residues interacting with 2h and 5m during unbinding.

Drug Atoms	Compd 2h			Drug Atoms	Compd 5m		
	Residues	Contact Area (Å^2)			Residues	Contact Area (Å^2)	
		COM[1]	COM[A392E]			COM[2]	
CL1	I261	6.2	7.4	C15	I261	9.1	
C1	P262	5.9	2.7	C15	P262	5.3	
CL1	K263	3.5	1.6	C12	A392	4.1	
CL1	I287	8.7	6.6	N3	P262	2.9	
O1	R127	4.1	2.9	O1	N264	2.8	
N2	P262	2.9	3.4	C16	R127	3.6	
C12	Y674		0.6	C17	K263		
				C17	I287		
**Diff**[a]	4.9			**Diff**[a]	3.7		

Table S8: Reported mutations for different class of compounds

Domain	Chemical class of active compounds	IC_{50} (µM)	EC_{50} (µM)	Reported mutations
Finger	Indole	0.02		F224S
	Imidazopyridine	--NA--		F224S
	Pyrimidin-amine	--NA--		F224Y
	Cyclic urea	--NA--		E291G
	Imidazopyridine	--NA--		E291G
	Arylazoenamine	28		E291G
	Benzimidazole	--NA--		I261M
	Arylazoenamine	28.0		I261M
	Benzimidazole	0.002		I261M
	Benzimidazole	--NA--		I261M+N264D
	Thiosemicarbazone	--NA--		N264D+A392E
	N-polycyclic	0.48	0.3	
	Phenantroline	12.0	6.0	
	Quinoline tricyclic	0.06	1.2	--NA--

Supplementary Figures

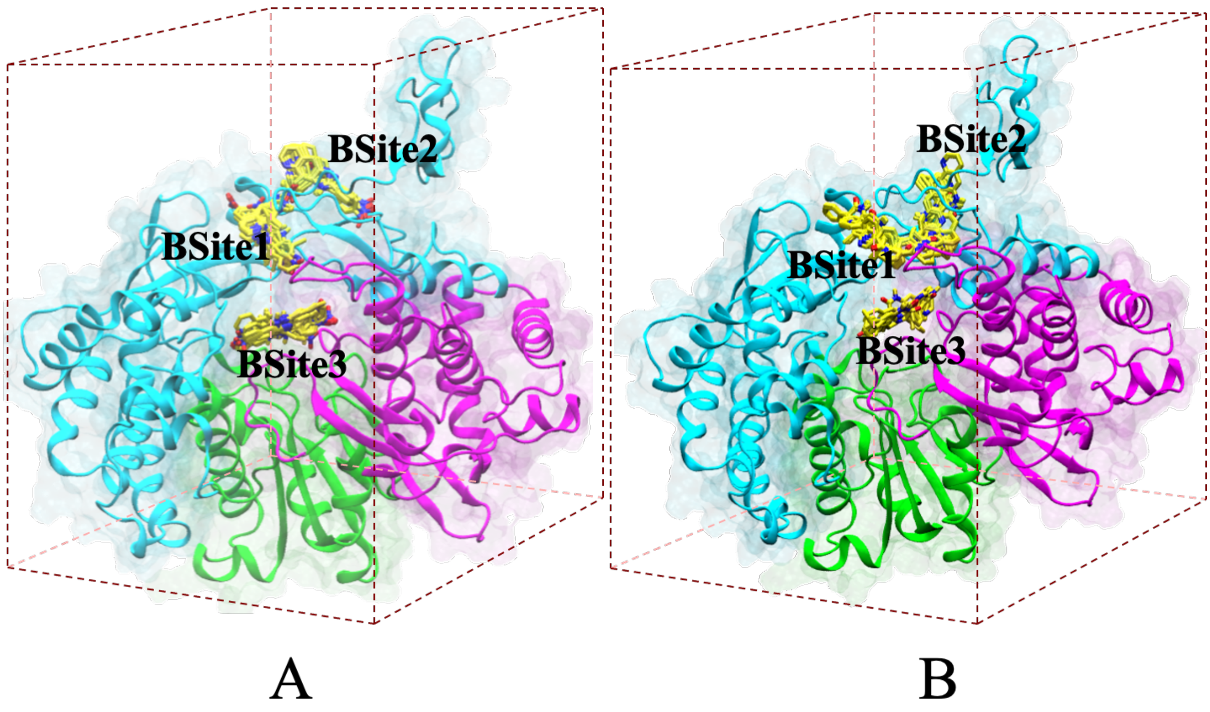

Figure S1: Blind docking reveals the clusters of (A) 2h and (B) 5m, respectively. The conformations of 2h and 5m are rendered in licorice (yellow)

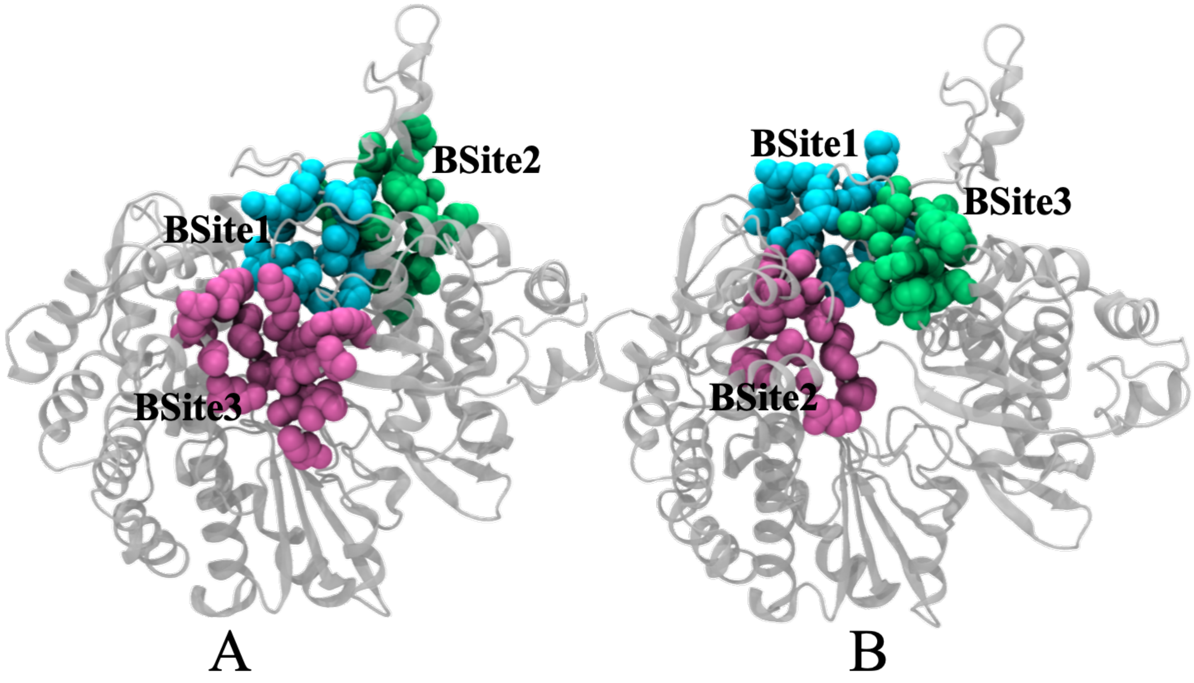

Figure S2: Molecular docking via AL reveals the clusters of (A) 2h and (B) 5m, respectively. The clusters at Bsite1, Bsite2 and Bsite3 are represented in vdW conformations and colored in cyan, green and mauve, respectively.

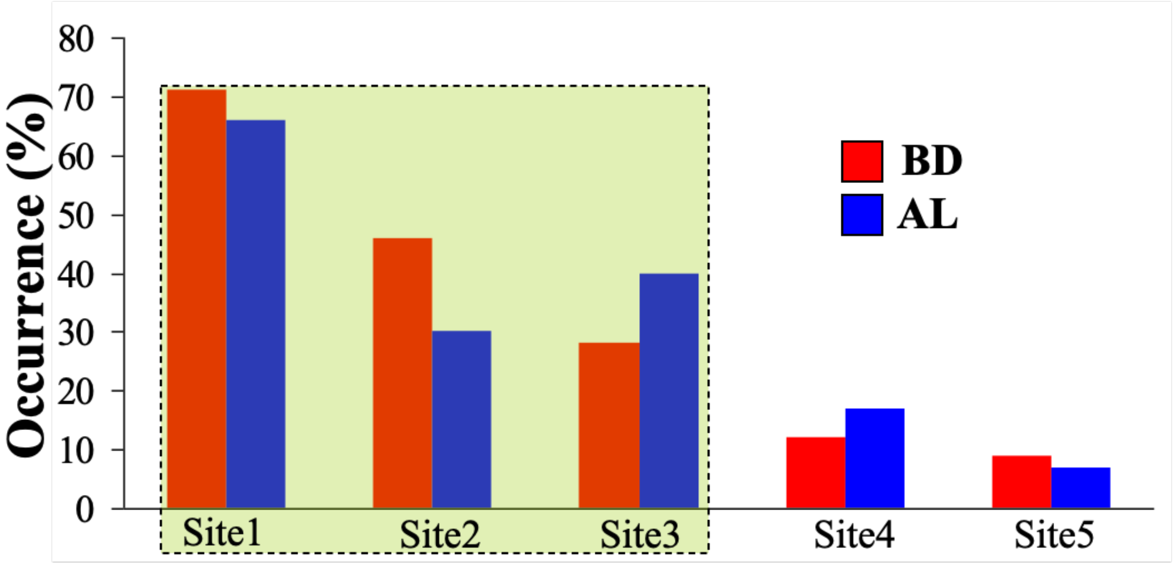

Figure S3: The focused ensemble-based docking (FD) on each conformers of protein targeting top five consensus sites identified through AL and BD steps. In particular top three sites have shown considerable binding affinity. The top three clusters of proteins (shown in inset), and were used for subsequent MD runs.

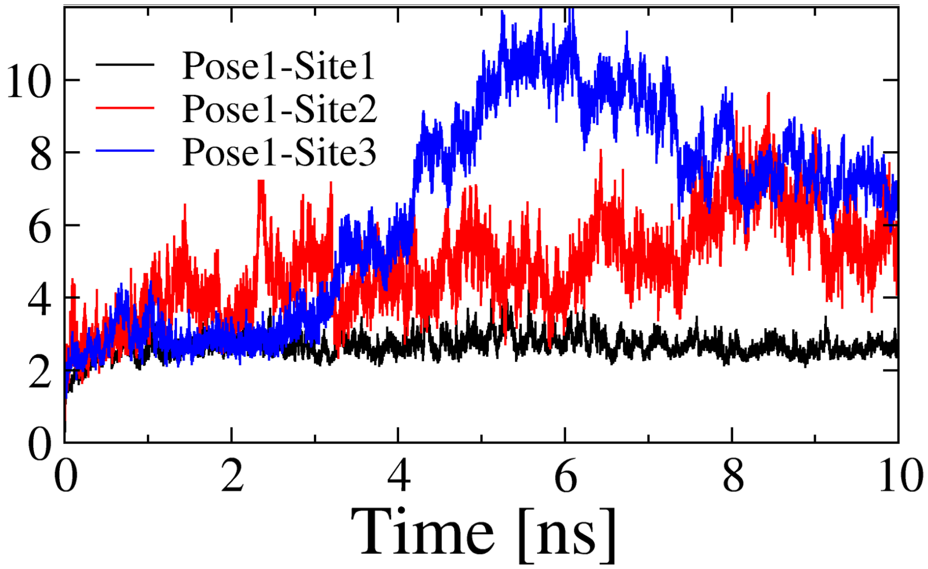

Figure S4: Validation of binding pose of 2h identified at Sites 1, 2 and 3 through MD simulation of 10 ns.

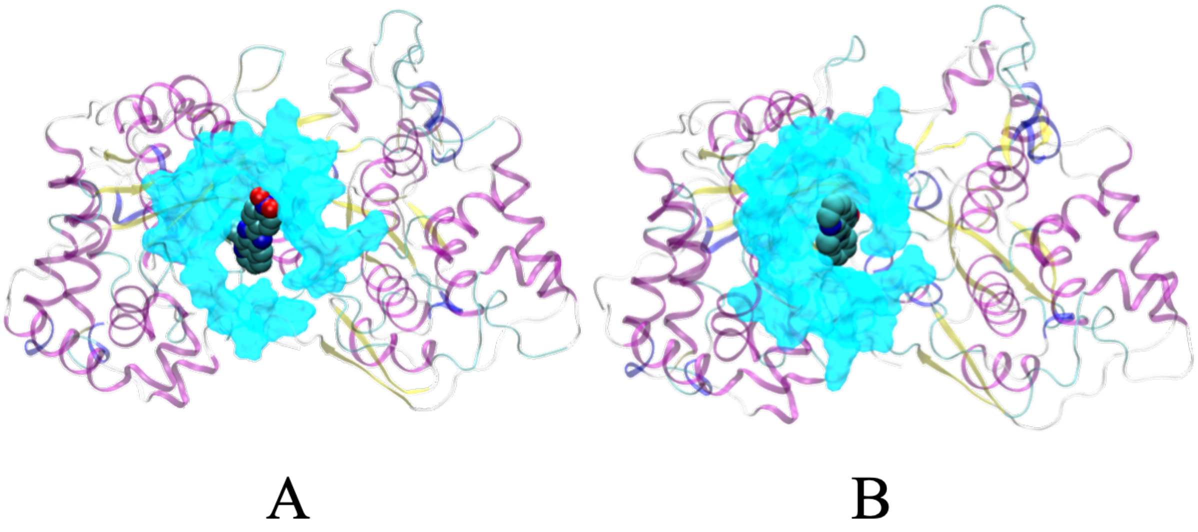

Figure S5: Occlusion of NTP entry channel upon binding of (A) 2h and (B) 5m

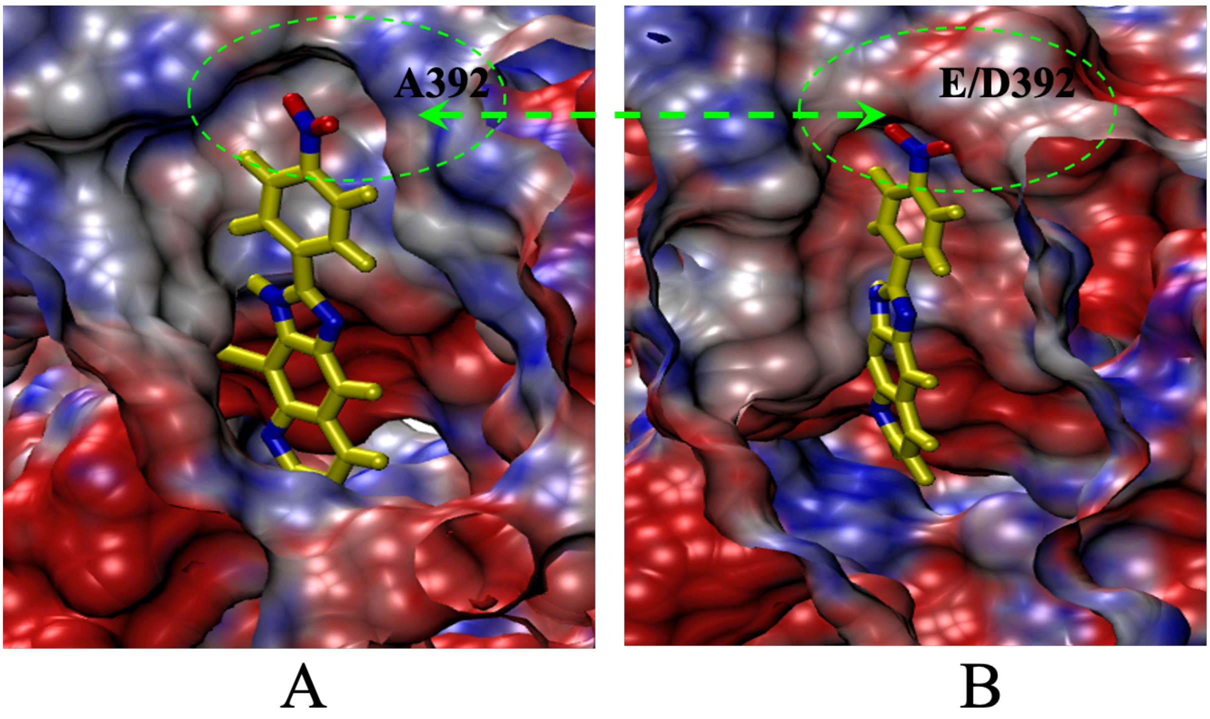

Figure S6: Electrostatic potential surface of (A) wild 2h and (B) mutant 2h

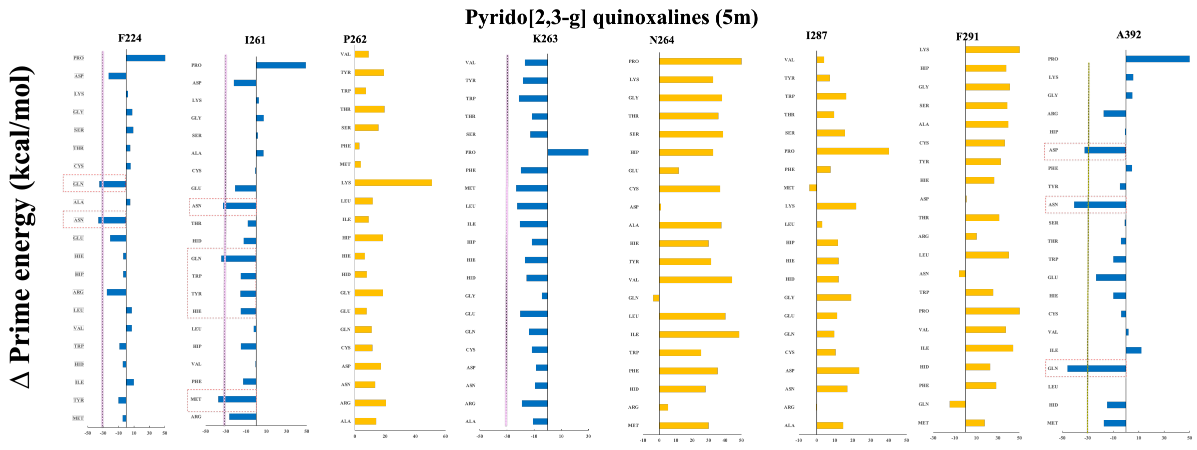

Figure S7: Mutational analysis on 5m
